# Phosphatase PTP-4 downregulates ATLN-1-mediated host defense against *Pseudomonas aeruginosa* infection in *Caenorhabditis elegans*

**DOI:** 10.3389/fphar.2026.1804420

**Published:** 2026-06-01

**Authors:** Jing Xie, Guojun Shi, Mo Wang, Yuwen Zhong, Yue Yuan, Lei Sun, Baoxue Ge, Ajing Xu

**Affiliations:** 1 Department of Clinical Pharmacy, Xinhua Hospital, School of Medicine, Shanghai Jiao Tong University, Shanghai, China; 2 The Third Affiliated Hospital of Sun Yat-sen University, Guangzhou, China; 3 Shanghai Frontiers Science Center of Drug Target Identification and Delivery, School of Pharmaceutical Sciences, Shanghai Jiao Tong University, Shanghai, China; 4 Shanghai Key Laboratory of Tuberculosis, Shanghai Pulmonary Hospital, Tongji University School of Medicine, Shanghai, China

**Keywords:** ATLN-1, baicalin, *Caenorhabditis elegans*, innate immune response, *Pseudomonas aeruginosa* (PA), PTP-4

## Abstract

**Aim:**

The p38 MAPK pathway governs the core innate immune response in *Caenorhabditis elegans* (*C. elegans*), yet the negative regulatory networks preventing immune overactivation—and their potential as therapeutic targets—remain elusive. We aimed to identify novel phosphatase checkpoints in host defense, elucidate their molecular mechanisms, and validate their druggability using small-molecule interventions.

**Methods:**

We performed a genome-wide RNAi screen of 166 phosphatase genes in *C. elegans* infected with *Pseudomonas aeruginosa* (PA14) to identify key regulators. The molecular mechanism was dissected using yeast two-hybrid screening, Co-IP, and mutagenesis assays. Furthermore, a small-molecule screen was performed to identify compounds capable of interfering with PTP-4 function, followed by Surface Plasmon Resonance (SPR), molecular docking, and structural biology approaches to characterize compound–PTP-4 interactions.

**Results:**

PTP-4 was identified as a critical negative regulator of immunity. Genetic deficiency of *ptp-4* significantly enhanced host resistance via hyperactivation of the p38 MAPK (PMK-1) pathway. Mechanistically, PTP-4 physically interacts with ATLN-1, an ER-shaping GTPase, through its phosphatase domain, thereby suppressing immune activation. Crucially, baicalin was identified through compound screening as an inhibitor of *P. aeruginosa* infection. Structural and biochemical analyses revealed a dual mechanism of action: baicalin directly binds to the PTP-4/ATLN-1 interface with high affinity (K_D_ = 28.69 μM) to allosterically disrupt their complex formation, while concurrently downregulating PTP-4 protein expression. This “dual-hit” inhibition mimics the genetic loss of *ptp-4*, effectively unleashing the innate immune response.

**Conclusion:**

Our study uncovers the PTP-4/ATLN-1 axis in the control of *P. aeruginosa* infection. Importantly, we provide the first proof-of-concept that this axis is a druggable target. By demonstrating that baicalin boosts host immunity through the specific dismantling of the PTP-4/ATLN-1 complex, we highlight a novel therapeutic strategy for combating bacterial infections.

## 1 Introduction

*Pseudomonas aeruginosa* (*P. aeruginosa,* PA), a clinically prevalent opportunistic pathogen, poses a severe threat to immunocompromised hosts, with infections in these patients carrying a high risk of progressing to life-threatening sepsis (Letizia et al., 2025; Botelho et al., 2019). In this process, the innate immune system serves as the frontline defense, rapidly mobilizing effector responses to neutralize the pathogen while priming adaptive immunity to establish long-term protection (Pradeu et al., 2024). Decoding the molecular regulators of these host-pathogen interactions offers transformative potential for via precision modulation of dysregulated pathways. Therefore, investigating the regulatory mechanisms underlying infection-induced innate immunity is of considerable importance.

**TABLE 1:** Primers used for quantitative real-time PCR.

Gene	Primer sequence (5′→3′)	Product size (bp)
*clec-60*	F:GATGCTGCTGATGATGCTGA	112
	R:CGATGATGATGATGATGATGGA	
*lys-2*	F: TGG​CTA​CTC​ACC​AAC​TTC​GA	102
	R: TGA​TGG​TAG​AGC​TGC​TCC​TC	
*gpd-2*	F: TCC​ACT​GTT​GGT​GTC​TGG​TC	101
	R: TGA​GCT​GGA​TGG​TGA​TCT​TCT	

*Caenorhabditis elegans* (*C. elegans*) is a premier model organism renowned for its anatomical simplicity, optical transparency, rapid development, and short life cycle. These attributes make it a pivotal model for dissecting conserved mechanisms of pathogen-host interactions, antimicrobial immunity, and therapeutic discovery (Tse-Kang et al., 2025; Martineau et al., 2021). Its innate immune system is characterized by evolutionarily conserved signaling pathways, including the IIS/IGF-1, TGF-β, p38 MAPK (PMK-1) cascades, which collectively orchestrate antimicrobial defenses and stress adaptation (Xiao et al., 2025; Poupet et al., 2020; Zheng et al., 2021).

The *P. aeruginosa-C. elegans* infection model has become a powerful platform for revealing critical regulators of innate immunity (Hajdu et al., 2024; Ermolaeva and Schumacher, 2014). Specifically, immune signaling is fine-tuned by a sophisticated phosphatase-kinase counterbalance, with the PMK-1/p38 MAPK pathway drives pathogen clearance (Li et al., 2018; Shivers et al., 2010; Xu et al., 2013). Conversely, the dual-specificity phosphatase VHP-1 dynamically suppresses both PMK-1 phosphorylation (p38 MAPK) and KGB-1 activation (JNK), exemplifying the phosphatase-kinase counterbalance that fine-tunes immune signaling (Mizuno et al., 2004; Egge et al., 2021). Despite these advances, the broader roles of phosphatases in immune regulation remain incompletely understood. Elucidating these spatiotemporal phosphorylation networks could reveal novel targets for modulating dysregulated immune responses, thereby providing new insights into phosphatase-targeted therapies for infectious diseases.

In this study, through a genome-wide RNAi screen of phosphatases in *C. elegans*, we identified PTP-4 as a critical negative regulator that suppresses host immunity via its interaction with the ER-shaping GTPase ATLN-1. Furthermore, by integrating small-molecule screening with structural biology approaches, we revealed that baicalin exerts potent anti-infective effects by targeting this specific axis. Taken together, our study not only identifies PTP-4 as a key regulator of *P. aeruginosa* infection and elucidates its underlying mechanism of action, but also uncovers a targetable compound, thereby providing new insights and therapeutic strategies for the study of host–pathogen interactions using *C. elegans*. More broadly, this work provides a proof-of-concept that pharmacological disruption of specific phosphatase complexes represents a viable approach for boosting innate immunity.

## 2 Materials and methods

### 2.1 Strains and cell lines

*Caenorhabditis elegans* were cultured on standard nematode growth medium (NGM) seeded with *E. coli* OP50. The following strains were obtained from the *Caenorhabditis* Genome Center (CGC): wild-type N2 Bristol, EG8919 [*ptp-4* (*oxTi977*)], and RB1127 [*atln-1* (*ok1144*)]. *E. coli* OP50 and HT115 (DE3) were obtained from the CGC. RNAi bacteria were kindly provided by Dr. Huanhu Zhu (Shanghai Tech University) and Dr. Chonglin Yang (Yunnan University). *P. aeruginosa* PA14 was provided by Dr. Bin Qi (Yunnan University). HEK293T cells were purchased from ATCC (Cat# CL-153).

Standard LB broth, NGM, and M9 buffer were prepared according to standard protocols (Zhang L. et al., 2023). Slow Killing (SK) Medium: NGM supplemented with 100 μg/mL 5-fluoro-2′-deoxyuridine (FUdR). Lysis Solution: A fresh 1:1 mixture of 10% sodium hypochlorite and 1 M NaOH.

Gravid adults were collected and treated with a lysis solution (1:1 mixture of 10% sodium hypochlorite and 1 M NaOH) to release eggs. The eggs were washed with M9 buffer and incubated overnight at 20 °C to obtain synchronized L1 larvae. For maintenance, worms were cultured on NGM plates seeded with *E. coli* OP50 at 20 °C (Zhang L. et al., 2023).

### 2.2 RNA-interference treatment

The RNAi clones targeting 166 genes were kindly provided by Dr. Chonglin Yang (Yunnan University). All bacterial clones were validated by Sanger sequencing. Bacteria were cultured in LB broth containing 50 μg/mL carbenicillin and seeded onto NGM plates supplemented with 2.4 mM IPTG. Plates were incubated overnight at room temperature to induce dsRNA expression (Lehner et al., 2006). Synchronized L1 (or L4) stage worms were then transferred to these plates for gene knockdown. A semi-quantitative Survival Index (SI) was employed to evaluate the primary screening hits. Candidates were assigned scores (0–3) based on the percentage of surviving worms relative to the L4440 control at the point of 100% control mortality: Score 1 (0%–30%); Score 2 (30%–60%); Score 3 (>60%). Hits that reproducibly achieved a score 2 (e.g., *ptp-4* and gene *no. 18*) were prioritized for subsequent high-resolution survival analysis. Detailed statistical parameters for the validated candidates are summarized in Supplementary Data 1.

### 2.3 *Pseudomonas aeruginosa* slow killing (SK) assay

Bacterial lawns were prepared by spreading overnight LB cultures of *P. aeruginosa* (or control *E. coli* OP50) onto SK plates. Plates were incubated at 37 °C for 24 h and then equilibrated at 25 °C for 24 h prior to use. Synchronized L1 larvae were reared on OP50 plates at 20 °C until the L4 stage. Fifty L4 worms were transferred to each assay plate (in triplicate) (Zhang L. et al., 2023). The survival assay was conducted at 25 °C. Worm survival was scored every 12 h for the infection group and every 48 h for the control group. Death was defined as the lack of movement upon mechanical stimulation. Worms that crawled off the plate, burrowed into the agar, or exhibited vulval rupture were recorded as censored on the day of the event and excluded from the subsequent lifespan analysis (Kirienko et al., 2014). Median survival times and P-values were calculated using the Log-rank (Mantel-Cox) test via GraphPad Prism 9.0.

### 2.4 Chemical reagents and preparation

Baicalin (Cat. No. B20570; Shanghai Yuanye Bio-Technology Co., Ltd., Shanghai, China) was dissolved in dimethyl sulfoxide (DMSO) to prepare a stock solution of 4 mg/mL. The solution was sterilized by filtration through a 0.45-μm syringe filter and stored at 4 °C. For all experiments, a vehicle control group containing 0.1% DMSO (v/v) was established.

### 2.5 *Pseudomonas aeruginosa* infection and rescue assay

Synchronized L4-stage nematodes were infected by culturing on *P. aeruginosa* PA14 lawns at 25 °C for 8 h. Following infection, the worms were harvested and washed three times with sterile M9 buffer to remove extracellular bacteria. Approximately 30–50 infected worms were transferred to each well of a 96-well plate containing M9 buffer supplemented with the indicated concentrations of baicalin. The plates were incubated at 25 °C, and survival was assessed at specified time intervals by counting live worms. All assays were performed in triplicate and repeated at least three times independently.

### 2.6 Cell culture

HEK293T cells were cultured in DMEM/High Glucose (Gibco) supplemented with 10% Fetal Bovine Serum (Biological Industries) and 1% Penicillin-Streptomycin (100 IU/mL penicillin, 100 μg/mL streptomycin). Cells were maintained in a humidified 5% CO_2_ atmosphere at 37 °C.

### 2.7 Bacterial colony-forming unit (CFU) assay

Intestinal bacterial loads were quantified as previously described. Briefly, infected worms were paralyzed with 25 mM levamisole to inhibit pharyngeal pumping and surface-sterilized with antibiotic-containing M9 buffer (1 mg/mL gentamicin and 1 mg/mL ampicillin) for 30 min. After washing, worms were homogenized in PBS using quartz sand. Serial dilutions of the lysates were plated onto LB agar containing 100 μg/mL rifampicin (to select for *P. aeruginosa* PA14) and incubated at 37 °C overnight. CFUs were counted from three independent experiments (Walker et al., 2022).

### 2.8 Western blot analysis

Worms were collected at the indicated time points post-infection or following drug treatment. Samples were washed with M9 buffer and lysed in RIPA buffer supplemented with protease and phosphatase inhibitors (Beyotime). Protein concentrations were determined using a BCA Protein Assay Kit. Lysates were resolved by SDS-PAGE and transferred to PVDF membranes. Membranes were blocked with 5% non-fat milk and incubated overnight at 4 °C with primary antibodies: anti-p-MAPK (1:1,000, Abclonal, ap1502), anti-PTP-4 (1:1,000, Abclonal, a22564), and anti-p-ATLN-1 (1:500, Abclonal, a12196). Loading controls included anti-GAPDH, anti-β-actin, or anti-β-tubulin (1:10,000, Abclonal). After incubation with HRP-conjugated secondary antibodies (CST), bands were visualized using a fluorescence imaging system and quantified with ImageJ software (Ma et al., 2023). All Western blot experiments were performed with at least three independent biological replicates (n = 3). Protein bands were normalized to loading controls, and statistical significance was determined by One-way (or Two-way) ANOVA followed by Tukey’s *post hoc* test for multiple comparisons. The original strip data has been archived and provided as Supplementary File 1.

### 2.9 Yeast two-hybrid screening

Yeast transformation was performed using standard protocols (Elmore et al., 2023). The full-length cDNA of *ptp-4* was cloned into the pBTM116 vector (LexA DNA-binding domain) as the bait. Bait protein expression was verified by Western blot analysis using an anti-T22 antibody. This construct was used to screen a *C. elegans* cDNA library. Positive interactors were identified by growth on selective media (SD/-Leu/-Trp/-His supplemented with 2.5 mM 3-AT) and confirmed by β-galactosidase lift assays. To ensure the reliability of the screening, the interaction strength was semi-quantitatively scored based on the intensity of the blue color in the X-gal assay (Score + to +++). Among the 9 reproducible positive clones identified, *atln-1* (Y54G2A.2) exhibited the most robust interaction signal (Score +++). Positive clones were subsequently identified by DNA sequencing. All interactions were confirmed by at least three independent yeast transformation events.

### 2.10 Protein purification

The coding sequence of *C. elegans ptp-4* was cloned into the pGEX-TEV vector and expressed in *E. coli* BL21 (DE3). Cells were grown at 37 °C until the OD reached 0.6–0.8, followed by induction with 0.5 mM IPTG at 16 °C for 16 h. GST-PTP-4 fusion proteins were purified using Glutathione Sepharose 4B beads (GE Healthcare) according to the manufacturer’s instructions. The protein was eluted with 20 mM reduced glutathione (pH 8.0) and dialyzed against PBS to remove free glutathione. Purity was assessed by Coomassie Blue staining.

### 2.11 Phosphatase activity assay

The phosphatase activity of PTP-4 protein (Sino Biological, China) was measured using a colorimetric assay based on p-nitrophenyl phosphate (p-NPP) as the substrate, following a well-established protocol (Ohsugi et al., 1997) with minor modifications. The reaction mixture (100 μL total volume) contained 50 mM HEPES (pH 7.2), 100 mM NaCl, 1 mM EDTA, 2 mM dithiothreitol (DTT), 0.01% bovine serum albumin (BSA), 20 nM PTP-4, and 2 mM p-NPP. Following incubation at 37 °C for 30 min, the absorbance was measured at 405 nm using a microplate reader. The amount of product (p-nitrophenol, p-NP) released was quantified by reference to a standard curve prepared with known concentrations of p-NPP. To verify the specificity of the assay, sodium orthovanadate (Na_3_VO_4_, 1 mM), a potent phosphatase inhibitor, was pre-incubated with PTP-4 for 10 min prior to substrate addition as a positive control for inhibition. A blank control consisting of the reaction buffer without enzyme was included to correct for non-enzymatic hydrolysis of p-NPP. All experiments were performed in triplicate with three independent replicates. p-NPP, sodium orthovanadate, and all buffer components were purchased from Sigma-Aldrich (United States). PTP-4 protein was obtained from Sino Biological (China).

### 2.12 Structural prediction and docking

Protein structures for PTP-4 (AF-P28192-F1) and ATLN-1 (AF-Q9BMU4-F1) were retrieved from the AlphaFold Protein Structure Database. Rigid-body protein-protein docking was simulated using the GRAMM server with high-resolution parameters, including a grid step of 1.1–1.7 Å and a rotational angle step of 10° for a global conformational search. Interface analysis was subsequently performed using PDBePISA.

### 2.13 Plasmid transfection and co-immunoprecipitation (Co-IP)

HEK293T cells were cultured in DMEM supplemented with 10% FBS and penicillin/streptomycin. Forty-eight hours post-transfection, cells were lysed in buffer containing 20 mM Tris (pH 7.5), 150 mM NaCl, 1 mM EDTA, and 1% Triton X-100, supplemented with protease inhibitors. Lysates were pre-cleared with Protein A/G agarose beads and incubated with anti-FLAG or anti-Myc antibodies overnight at 4 °C. Immunocomplexes were captured by beads, washed four times, and eluted by boiling in SDS loading buffer for Western blot analysis.

### 2.14 Fluorescence confocal microscopy

FLAG-PTP-4 (300 ng) and Myc-ATLN-1 (300 ng) plasmids were transfected into HEK293T cells seeded on poly-L-lysine-coated coverslips in 10 cm dishes. At 36 h post-transfection, cells were treated with 1 mg/mL *P. aeruginosa* PA14 total lysate or PBS for 2 h, then fixed with 4% paraformaldehyde (25 °C, 20 min), permeabilized with 0.1% Triton X-100 (10 min), and blocked with 5% BSA (25 °C, 1.5 h). Primary antibodies were incubated for 4 h at 25 °C, followed by Alexa Fluor 594 goat anti-rabbit IgG and Alexa Fluor 488 goat anti-mouse IgG (1:1,000 in 5% BSA) for 1 h. Slides were mounted with DAPI-containing medium and imaged using a Carl Zeiss LSM710 confocal microscope (63 × 1.4 NA oil immersion objective). Colocalization was quantified via ImageJ software.

### 2.15 Immunoprecipitation

Immunoprecipitation was performed as previously described Briefly, co-transfected HEK293 cells were lysed in 2% Triton X-100 lysis buffer [20 mM Tris (pH 7.5), 150 mM NaCl, 1 mM EDTA] supplemented with protease inhibitor cocktail (Roche). Protein concentration was determined using the Enhanced BCA Protein Assay Kit (Beyotime Biotechnology). Lysates were pre-cleared with Protein A + G Agarose beads (Beyotime Biotechnology) for 1 h, then incubated with target antibodies overnight at 4 °C with rotation. Immunoprecipitates were washed four times with IP lysis buffer, eluted in 2× SDS-PAGE loading buffer (95 °C, 5 min), and resolved by SDS-PAGE (Ma et al., 2023).

### 2.16 Virtual screening of small-molecule inhibitors

A multi-tier virtual screen was conducted using Discovery Studio v3.5 (Accelrys, United States) to identify potential PTP-4 inhibitors from the COlleCtion of Open Natural produCTs (COCONUT) database (715,344 molecules; Sorokina and Steinbeck, 2020) (Zhao et al., 2023). The library was sequentially filtered for chemical stability, toxicity, and drug-likeness (Lipinski’s Rule of Five and ADME (Absorption, Distribution, Metabolism, and Excretion) properties), narrowing the candidates to 171,623. After clustering analysis to ensure structural diversity, 1,054 representative molecules were subjected to molecular docking against the PTP-4 active site. Baicalin was identified as the top candidate based on its superior binding score and established bioactivity in *C. elegans* models. The docking scores of the candidates are detailed in Supplementary Data 2 (Excel file).

### 2.17 Surface plasmon resonance (SPR) analysis

The binding kinetics between baicalin and PTP-4 protein (Sino Biological, China) were determined using a Biacore T200 system (Cytiva, UK) at 25 °C. PTP-4 was immobilized onto a CM5 sensor chip (Cytiva, UK) via amine coupling. pH scouting experiments identified 10 mM sodium acetate (pH 4.5) as the optimal condition for ligand immobilization. The chip surface was activated with a mixture of NHS/EDC at a flow rate of 10 μL/min for 7 min. PTP-4 diluted in 10 mM sodium acetate (pH 4.5) was then injected until an immobilization level of approximately 6,000 resonance units (RU) was achieved. Residual activated groups were blocked with 1 M ethanolamine (pH 8.5). HBS-EP+ was used as the running buffer throughout the experiments. Baicalin (≥98% purity, Sigma-Aldrich, United States) was serially diluted two-fold with running buffer to concentrations ranging from 0.12 to 10 μM. Samples were injected at a flow rate of 30 μL/min with an association time of 120 s and a dissociation time of 180 s. The sensor chip surface was regenerated after each cycle with 10 mM glycine-HCl (pH 2.0) at 30 μL/min for 30 s. Data were analyzed using Biacore T200 Evaluation Software (version 3.0) with a 1:1 Langmuir binding model to calculate the association (*k*_*a*_) and dissociation (*k*_*d*_) rate constants, from which the equilibrium dissociation constant (*K*_*D*_) was derived. Background noise and bulk effects were minimized by double-referencing (subtracting both the reference flow cell and the zero-concentration buffer injection). All measurements were performed in triplicate.

### 2.18 Sequence alignment

Multiple sequence alignment of the PTPase domains from various human PTP family members and the *C. elegans* ortholog PTP-4 was performed using the CLUSTALW program (https://www.genome.jp/tools-bin/clustalw). The alignment result was then visualized and annotated with secondary structure elements using the ESPript 3.0 server (http://espript.ibcp.fr/ESPript/cgi-bin/ESPript.cgi). We have clarified that this represents a sequence alignment annotated with predicted secondary structure elements derived from the ESPript server, rather than a true structural alignment based on PDB coordinates. Accordingly, we have revised the description in the main text and figure legends to accurately reflect this methodology.

### 2.19 Quantitative real-time PCR (qRT-PCR)

Total RNA was extracted from synchronized L4-stage *C. elegans* (wild-type N2 and *ptp-4* (*ok1411*) mutants) following 12 h of exposure to either OP50 (control) or PA14 (infection) using the RNeasy Mini Kit (Qiagen, Germany). The concentration and purity of the isolated RNA were determined by measuring the *OD*_*260*_ */OD*_*280*_ ratio with a NanoDrop One spectrophotometer (Thermo Fisher Scientific, United States).

Subsequently, 1 μg of total RNA was reverse-transcribed into cDNA using M-MuLV reverse transcriptase. Quantitative real-time PCR (qRT-PCR) was performed on an ABI 7500 Real-Time PCR System using SYBR Green Master Mix. The relative expression levels of the target antimicrobial genes (*clec-60* and *lys-2*) were calculated using the 2^−ΔΔCt^ method and normalized to the internal reference gene *gpd-2*.

### 2.20 Statistical analysis

Data are expressed as mean ± SEM from at least three independent experiments. Statistical analyses were performed using GraphPad Prism 9.5.1. Survival data were analyzed via Kaplan-Meier curves and Log-rank tests, with censored animals (e.g., vulval rupture) excluded. Group comparisons were conducted using one-way or two-way ANOVA followed by Tukey’s *post hoc* test. P values <0.05 were considered statistically significant. All raw experimental records, including screening scores and WB densitometry, are available in the Source Data files.

## 3 Results

### 3.1 Phosphatase PTP-4 acts as a negative regulator of host immunity in *Caenorhabditis elegans* during PA14 infection

**FIGURE 1:**
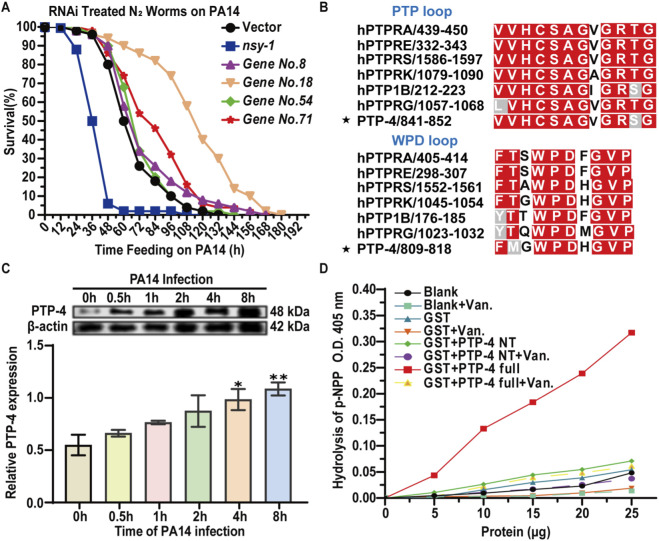
The conserved phosphatase PTP-4 acts as a negative regulator of host immunity in *Caenorhabditis elegans* during PA14 infection. **(A)** RNAi technology screens for candidate protein phosphatase genes (Gene. 71 (Gene, *ptp-4*)) that have effects on PA*14* infection. **(B)** The PTPase domain sequences exhibit similarity. **(C)** *Pseudomonas aeruginosa (*PA*14)* infection results in upregulated expression of the PTP-4 protein in wild-type nematodes. Relative quantification of the WB results, normalized to β-actin. **(D)** The purified full-length PTP-4 GST fusion expression protein possesses phosphatase activity. Data are representative of three independent biological replicates (n = 50 per group), with *P*-values determined by the Log-rank (Mantel-Cox) test **(A)**. Data represent mean ± SEM of n = 3 independent biological replicates. Group comparisons were conducted using one-way ANOVA followed by Tukey’s *post hoc* test. *P* < 0.05 was considered statistically significant (*P < 0.05, **P < 0.01, ***P < 0.001, ****P < 0.0001) **(C,D)**.

To identify protein phosphatases that negatively regulate innate immunity, we performed a targeted RNAi screen of 166 phosphatase genes in *C. elegans*. From this screen, we identified the 71st candidate gene, *ptp-4*, as a top hit; its knockdown significantly extended host survival following *P. aeruginosa* PA14 infection, exhibiting a protective efficacy comparable to that of the well-characterized negative regulator *vhp-1* (Figure 1A).

Given that the biological function of *ptp-4* remains poorly characterized, we first analyzed its sequence features to predict its molecular role. Multiple sequence and structural alignments revealed that the PTP-4 protein harbors a highly conserved intracellular PTPase domain homologous to human PTP family members (Figure 1B; Supplementary Figures S1-S3), implying that PTP-4 possesses intrinsic phosphatase activity.

Based on this observation, we further examined whether *ptp-4* is regulated during PA14 infection and found that PA14 challenge markedly induced *ptp-4* expression at 4 h and 8 h post-infection (Figure 1C). To directly test the predicted enzymatic function, we performed *in vitro* phosphatase activity assays, which confirmed that *ptp-4* indeed exhibits phosphatase activity (Figure 1D). Collectively, these results indicate that PTP-4 is a functional phosphatase and suggest that it may act as a key regulator in the host response to bacterial infection.

### 3.2 PTP-4 negatively regulated innate immunity and PMK-1 phosphorylation

**FIGURE 2:**
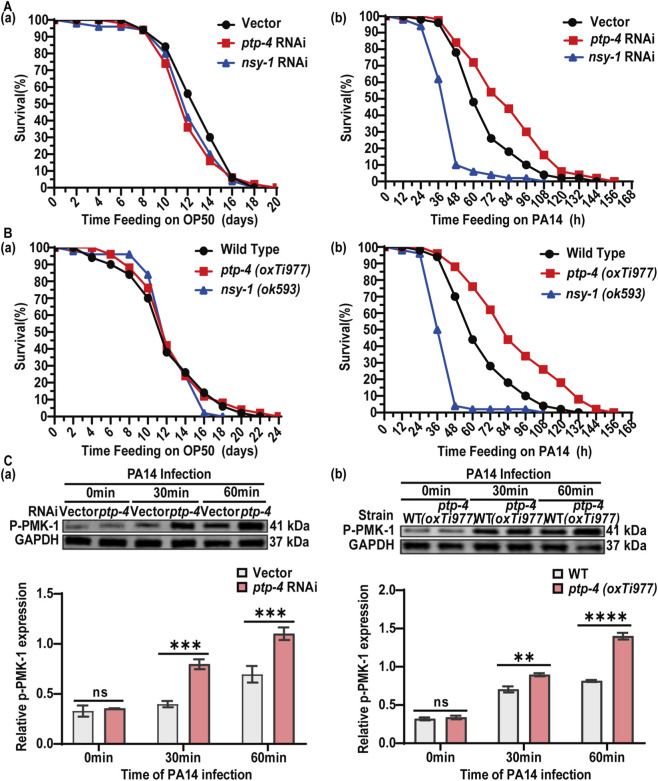
PTP-4 negatively regulates innate immunity and PMK-1 phosphorylation. **(A)** RNAi of *Caenorhabditis elegans ptp-4* (Gene No. 71) enhances resistance against *Pseudomonas aeruginosa* infection. (a) Survival of wild-type worms post-RNAi on *OP50* plates; (b) Survival of wild-type worms post-RNAi on PA*14* plates. **(B)** *ptp-4* mutation increases the worms' resistance to PA*14* infection. (a) Survival of *ptp-4* mutant worms on *OP50* plates; **(B)** Survival of *ptp-4* mutant worms on PA*14* plates. **(C)** PTP-4 regulates *Pseudomonas aeruginosa* infection-induced P-PMK-1 expression. (a) Increased phosphorylation level of PMK-1 in wild-type worms post-PA*14* infection following *ptp-4* gene RNAi; (b) Increased phosphorylation level of PMK-1 in *ptp-4* mutant worms post-PA*14* infection. Relative quantification of WB results, normalized to β-actin. Data are representative of three independent biological replicates (n = 50 per group), with *P*-values determined by the Log-rank (Mantel-Cox) test **(A,B)**. Data represent mean ± SEM of n = 3 independent biological replicates. Group comparisons were conducted using one-way ANOVA followed by Tukey’s *post hoc* test. *P* < 0.05 was considered statistically significant (*P < 0.05, **P < 0.01, ***P < 0.001, ****P < 0.0001) **(C)**.

To validate the findings from our RNAi screen, we examined the susceptibility of the genetic deletion mutant *ptp-4* (*oxTi977*). Survival assays confirmed that loss of *ptp-4*, achieved either through RNAi knockdown or genetic mutation, conferred significant resistance to *P. aeruginosa* PA14 infection compared to wild-type (N2) and empty vector controls (Figures 2A,B). In contrast, mutants of the upstream p38 MAPKKK nsy-1, included as a susceptibility control, succumbed rapidly to infection. Crucially, the extended survival observed in *ptp-4* deficient worms was pathogen-specific and not due to general longevity, as no significant differences in lifespan were observed when these strains were fed non-pathogenic *E. coli* OP50 (Figures 2A,B).

Given that the p38 MAPK pathway is the central regulator of innate immunity in *C. elegans*, we next investigated whether PTP-4 modulates this signaling cascade. Western blot analysis revealed that the phosphorylation levels of PMK-1 were markedly increased in *ptp-4* deficient worms at 30 and 60 min post-infection compared to wild-type controls (Figure 2C). To determine if this increased signaling translates into a specific immune output, we examined the expression of PMK-1-regulated effectors. While *clec-60* and *lys-2* levels remained low and comparable under basal conditions (OP50), their induction upon PA14 challenge was significantly amplified in *ptp-4* mutants compared to wild-type controls (Supplementary Figure S4). This pathogen-dependent hyper-activation confirms that PTP-4 specifically moderates the magnitude of the innate immune response during pathogen challenge rather than inducing general stress. These results indicate that PTP-4 functions as a negative regulator of innate immunity by restricting the activation of the p38 MAPK pathway.

### 3.3 Identification of ATLN-1 as a downstream effector of PTP-4

**FIGURE 3:**
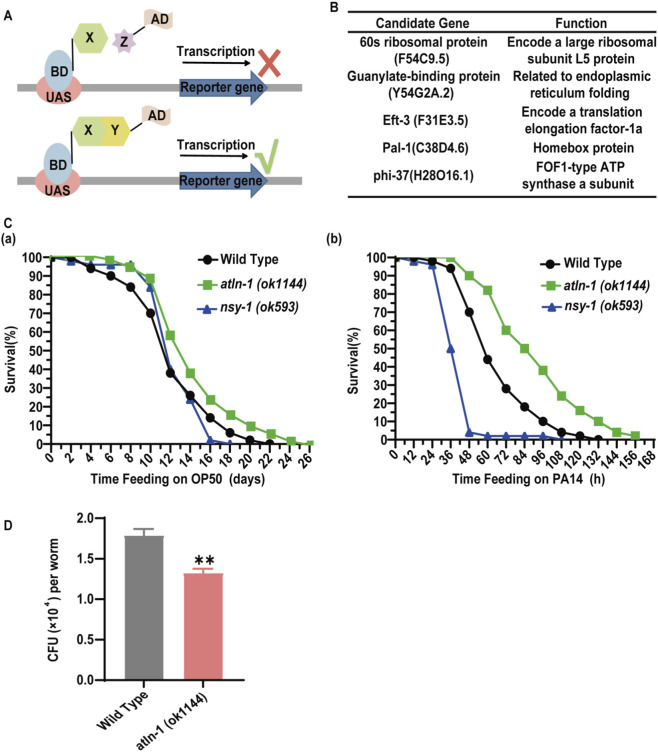
Identification of ATLN-1 as a Downstream Effector of PTP-4. **(A)** Schematic representation of the principle of the yeast two-hybrid technique. **(B)** Target gene clones interacting with the *ptp-4* gene screened by our laboratory using the yeast two-hybrid technique. **(C)** Variations in resistance to *Pseudomonas aeruginosa* infection in *atln-1* mutants. (a) Survival of *atln-1* mutant worms on OP50 plates; (b) Survival of *atln-1* mutant worms on PA*14* plates. **(D)** Reduced accumulation of CFU in the gut of *atln-1* mutants following *Pseudomonas aeruginosa* infection. Data are representative of three independent biological replicates (n = 50 per group), with P-values determined by the Log-rank (Mantel-Cox) test **(C)**. Data represent mean ± SEM of n = 3 independent biological replicates. Group comparisons were conducted using one-way ANOVA followed by Tukey’s *post hoc* test. *P* < 0.05 was considered statistically significant (*P < 0.05, **P < 0.01, ***P < 0.001, ****P < 0.0001) **(D)**.

To elucidate the molecular mechanism underlying PTP-4-mediated immune suppression, we performed a yeast two-hybrid (Y2H) screen to identify potential binding partners. After confirming bait expression, this screening identified ATLN-1 (encoded by sequence Y54G2A.2) as a direct interactor of PTP-4 (Figures 3A,B). Among the 9 candidates identified, ATLN-1 showed the strongest interaction based on X-gal scoring (Supplementary Table S3). While other candidates were also identified, such as a 60S ribosomal protein (F54C9.5), we prioritized ATLN-1 for further characterization. This choice was based on the fact that riboso proteins are frequently encountered as non-specific hits in Y2H assays. Moreover, since PA14 exotoxins directly target the host translation machinery, the potential confounding effects of ribosomal components might obscure the specific immunomodulatory role of the PTP-4 pathway.

As the effect of ATLN-1 on *P. aeruginosa* infection has never been studied, we first subjected *atln-1* (*ok1144*) deletion mutants to *P. aeruginosa* infection assays. Consistent with a role in negative regulation, *atln-1* mutants displayed a robust resistance phenotype, characterized by significantly prolonged survival and reduced intestinal bacterial burden (CFUs) compared to wild-type controls (Figures 3C,D). Notably, this resistance phenotype mirrors that of *ptp-4* mutants, suggesting that these two proteins function in the same genetic pathway. As observed with *ptp-4*, the extended lifespan of *atln-1* mutants was specific to pathogen challenge, as survival on standard *E. coli* OP50 was unaffected. Taken together, these findings identify ATLN-1 as a critical mediator of antimicrobial immunity and suggest that PTP-4 may exert its immunomodulatory effects via the regulation of the ATLN-1 axis.

### 3.4 Mapping the interaction domains of PTP-4 and ATLN-1

**FIGURE 4:**
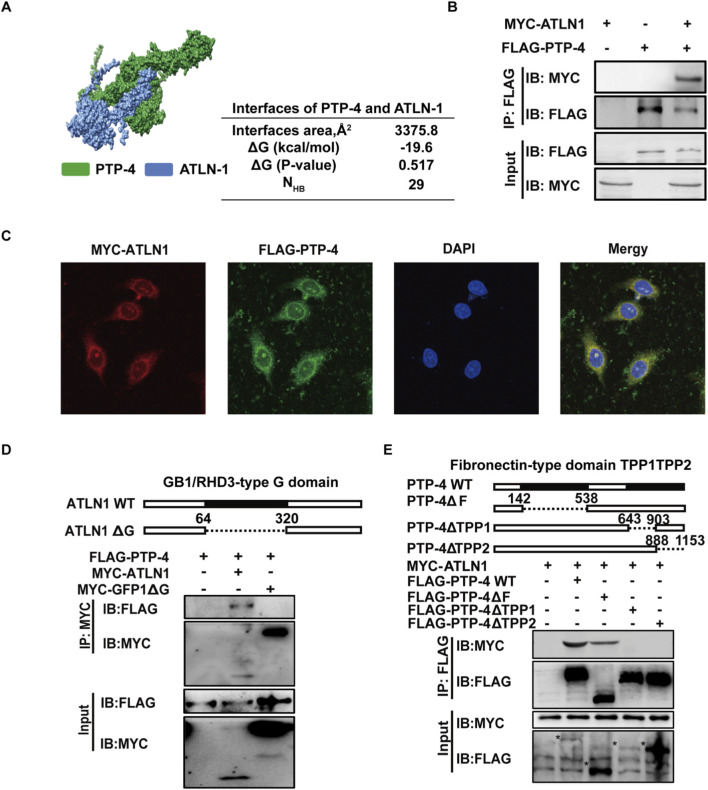
Mapping the Interaction Domains of PTP-4 and ATLN-1 **(A)** Overall density map of molecular docking between PTP-4 and ATLN-1, with green representing PTP-4 and blue representing ATLN-1. **(B)** Interaction between PTP-4 and ATLN-1 proteins (Co-IP). **(C)** Interaction between PTP-4 and ATLN-1 proteins (Confocal). **(D)** Transfection of HEK293T cells with FLAG-PTP4, MYC-ATLN-1, or MYC-ATLN-1 mutants, followed by co-immunoprecipitation using a MYC antibody to detect the interactions between PTP4 and different mutants of ATLN-1. **(E)** Interaction of PTP4 with ATLN-1 through the TPP1 and TPP2 domains. Transfection of HEK293T cells with MYC-ATLN-1, FLAG-PTP4, or FLAG-PTP4 mutants, and subsequent co-immunoprecipitation using a FLAG antibody to detect the interactions between mutants of ATLN-1 and PTP4. Data are representative of three independent experiments with similar results **(C–E)**.

To gain structural insights into the PTP-4/ATLN-1 complex, we first performed rigid-body molecular docking simulations using the GRAMM server. The simulation utilized the Fast Fourier Transform (FFT) shape complementarity principle to conduct a global search for the intermolecular interface. The computational model predicted multiple potential contact interfaces between the two proteins (Figure 4A; Supplementary Figure S5).

To further confirm the physical interaction between PTP-4 and ATLN-1, we expressed the *C. elegans*-derived cDNAs of these two proteins in HEK293T cells. Although HEK293T is a mammalian cell line, it provides a superior biochemical environment with high-fidelity protein synthesis and post-translational modification machinery, making it an ideal heterologous expression system to study the intrinsic binding affinity of nematode proteins. Using this approach, we performed co-immunoprecipitation (Co-IP) assays in cells co-expressing FLAG-tagged PTP-4 and Myc-tagged ATLN-1. Our results confirmed a robust physical association between these two nematode proteins (Figure 4B). Consistently, confocal microscopy analysis revealed extensive intracellular colocalization of PTP-4 and ATLN-1 (Figure 4C), further validating the spatial proximity required for their interaction.

Furthermore, to pinpoint the specific domains responsible for this interaction, we generated a series of truncation mutants: ATLN-1 variants lacking the GTPase domain (ATLN-1ΔG) and PTP-4 variants lacking the FERM domain (PTP-4ΔF) or the phosphatase domains (PTP-4ΔPTP1/ΔPTP2). Co-IP analysis demonstrated that deletion of either the ATLN-1 GTPase domain or the PTP-4 catalytic phosphatase domains (PTP1/2) completely abolished the interaction (Figures 4D,E). Collectively, these findings indicate that the binding is specific and relies critically on the GTPase domain of ATLN-1 and the phosphatase domains of PTP-4.

### 3.5 Baicalin confers protection against *Caenorhabditis elegans* during *Pseudomonas aeruginosa* PA14 infection

**FIGURE 5:**
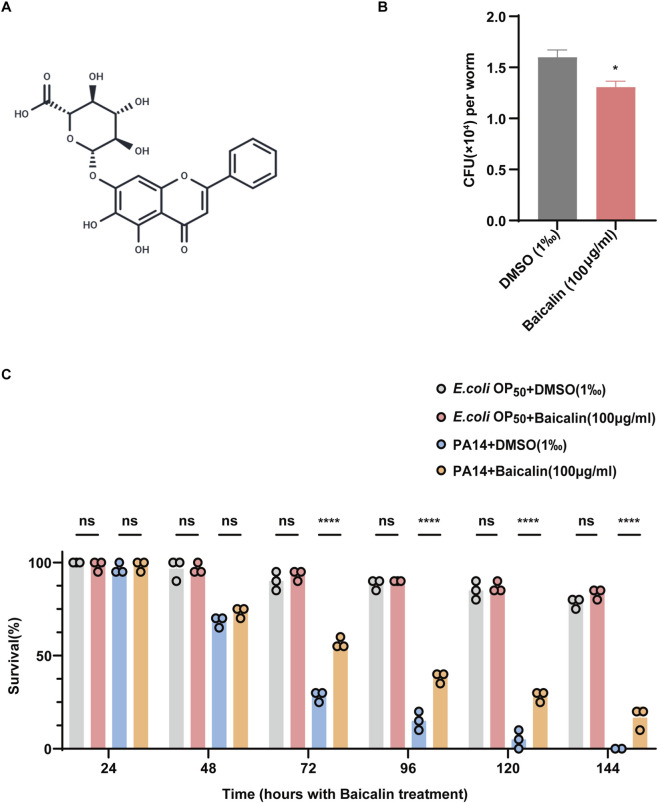
Baicalin protects *Caenorhabditis elegans* against *Pseudomonas aeruginosa* PA14 infection. **(A)** Chemical structure of baicalin. **(B)** Quantification of PA14 colonization in the intestine of *Caenorhabditis elegans* treated with or without baicalin (100 μg/mL). CFU counts were performed at 24 h post-infection. **(C)** Biosafety evaluation and survival analysis of *Caenorhabditis elegans.* Left bars show the survival percentage under PA14 infection with or without baicalin treatment; right bars show the effect of baicalin on the lifespan under non-pathogenic conditions (fed *E. coli* OP50). Data are representative of three independent biological replicates (n = 50 per group). Group comparisons were conducted using two-way ANOVA followed by Tukey’s *post hoc* test. *P* < 0.05 was considered statistically significant (*P < 0.05, **P < 0.01, ***P < 0.001, ****P < 0.0001) **(B,C)**.

Based on our findings that PTP-4 regulates *P. aeruginosa* infection, we performed a systematic virtual screen to identify small-molecule compounds targeting this axis. Starting from the COCONUT database (715,344 molecules), we employed a multi-stage filtering pipeline—including toxicity filtering, Lipinski’s rules, ADME screening, and clustering analysis—followed by molecular docking against the PTP-4 protein. From this extensive screen, the flavonoid monomer Baicalin (ID: CNP0175313.1) emerged as a top candidate. As shown in Figure 5A (or Supplementary Figure S6), the binding affinity of this scaffold (approximately −10.1 kcal/mol) represents an extreme outlier in the overall distribution, ranking within the top 0.1% of all screened compounds. This computational prediction is further supported by our previous laboratory findings, which established the potent anti-inflammatory efficacy and biosafety of Baicalin in *C. elegans* (Ma et al., 2020). Consequently, we selected Baicalin for further functional validation.

Before exploring its molecular interaction with PTP-4, we first evaluated its therapeutic efficacy in the *C. elegans*-*P. aeruginosa* infection model. Colonization analysis revealed that baicalin treatment significantly suppressed the accumulation of *P. aeruginosa* PA14 within the *C. elegans* intestinal lumen (Figure 5B), suggesting an enhancement of host defense. Paralleling this reduction in bacterial burden, survival assays demonstrated that administration of baicalin significantly extended the lifespan of PA14-infected nematodes compared to untreated controls (Figure 5C). Importantly, baicalin treatment did not significantly alter the survival of uninfected worms (*E. coli* OP50 group) within the same time window (Figure 5C). This confirms that the improved survival observed is due to enhanced resistance against PA14 infection rather than a general extension of host longevity or non-specific effects.

### 3.6 Baicalin targets the PTP-4/ATLN-1 interaction to potentially modulate host defense

**FIGURE 6:**
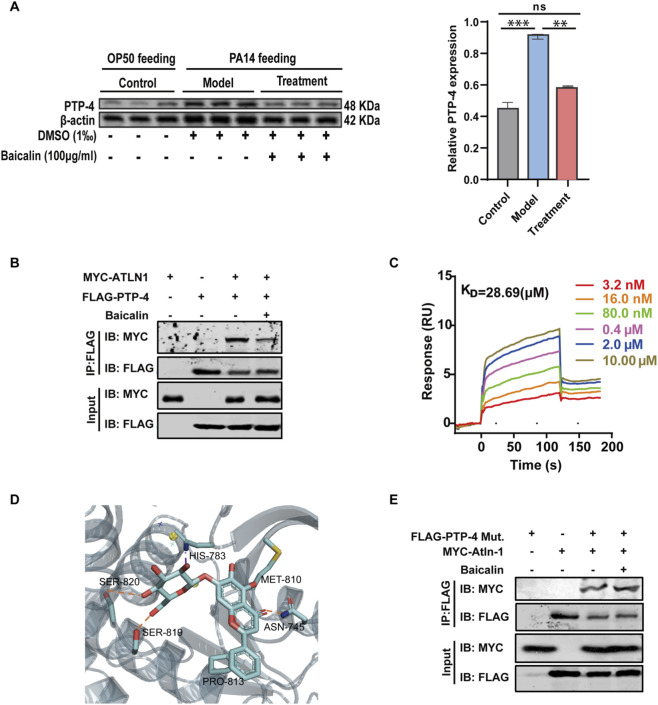
Baicalin promotes the innate immune response of *Caenorhabditis elegans* by disrupting the PTP-4/ATLN-1 interaction. **(A)** Western blot analysis showing the reversal of infection-induced PTP-4 upregulation in wild-type nematodes treated with baicalin following PA14 infection. **(B)** Co-immunoprecipitation (Co-IP) assays in HEK293T cells co-transfected with FLAG-PTP-4 and MYC-ATLN-1 plasmids. Immunoblotting with anti-FLAG antibody demonstrates that baicalin disrupts the interaction between PTP-4 and ATLN-1. **(C)** Surface Plasmon Resonance (SPR) analysis characterizing the direct binding affinity between baicalin and purified recombinant PTP-4 protein. **(D)** Molecular docking simulation illustrating the binding mode between baicalin and PTP-4. The PTP-4 protein structure is shown in gray, and baicalin is represented as a stick model. Key interacting residues are highlighted. **(E)** Validation of the binding site using PTP-4 mutants. HEK293T cells were transfected with MYC-ATLN-1 and mutant FLAG-PTP-4 constructs. Co-IP analysis indicates that mutations in the binding interface abolish the effect of baicalin on the PTP-4/ATLN-1 interaction. Data represent mean ± SEM of n = 3 independent biological replicates. Group comparisons were conducted using one-way ANOVA followed by Tukey’s *post hoc* test. *P* < 0.05 was considered statistically significant (*P < 0.05, **P < 0.01, ***P < 0.001, ****P < 0.0001) **(A,B,E)**.

Having established the protective efficacy of baicalin *in vivo*, we next sought to elucidate its effect on PTP-4. Western blot analysis showed that baicalin treatment significantly reduced PTP-4 protein levels in a dose-dependent manner (Figure 6A). Co-immunoprecipitation (Co-IP) assays in HEK293T cells demonstrated that baicalin disrupts the physical association between PTP-4 and ATLN-1 (Figure 6B). Consistent with this, Surface Plasmon Resonance (SPR) analysis confirmed a direct, high-affinity binding between baicalin and purified PTP-4, with an equilibrium dissociation constant (K_D_) of 28.69 μM (Figure 6C). Combined with the Co-IP results, these findings indicate that baicalin interacts with PTP-4 and interferes with the formation of the PTP-4/ATLN-1 complex.

Furthermore, computational docking simulations provided structural insights into this interaction, suggesting that baicalin is predicted to interact with the PTP-4 binding pocket through four hydrogen bonds (ASN745, MET810, SER819, SER820), hydrophobic interactions (PRO813), and a salt bridge (HIS783) (Figure 6D). In addition, we generated PTP-4 mutants lacking these key residues and found that these mutations abrogated the baicalin-mediated inhibition of the PTP-4/ATLN-1 interaction (Figure 6E). Collectively, these findings support a model where baicalin exerts its effects by exerting its immunomodulatory effects through a dual mechanism: directly binding to PTP-4 to disrupt the PTP-4/ATLN-1 complex and concurrently suppressing PTP-4 protein expression, thereby potentiating host innate immunity.

## 4 Discussion

In this study, we screened a genome-wide RNAi library of phosphatases and identified the receptor-like protein tyrosine phosphatase PTP-4 as a critical negative regulator of innate immunity in *C. elegans.* We demonstrated that PTP-4 deficiency significantly enhanced host resistance to *P. aeruginosa* (PA14) infection. Mechanistically, our data indicates that PTP-4 suppresses the immune response by negatively regulating the phosphorylation of the p38 MAPK (PMK-1), thereby preventing excessive immune activation. Consistent with the p38 MAPK pathway being the central driver of nematode immunity, the elevated p-PMK-1 levels in *ptp-4* mutants underscore a PMK-1-dependent regulatory axis. Therefore, the resistance phenotypes observed here are expected to be fundamentally dependent on functional PMK-1, which serves as the indispensable executor for downstream immune effector genes.

The protein tyrosine phosphatase (PTPase) family is a key regulator of inflammatory responses (Elmore et al., 2023). In *C. elegans*, the dual-specificity phosphatase VHP-1 was the first identified to negatively regulate the phosphorylation of PMK-1 in the p38 MAPK pathway under heavy metal stress. Meanwhile, VHP-1 also negatively regulates the phosphorylation of KGB-1 MAPK in the c-JUN-terminal kinase (JNK) signaling pathway, playing a critical role in hypersensitivity to heavy metal stress (Mizuno et al., 2004; Egge et al., 2021). While VHP-1 is a known regulator of the p38 pathway, it remains possible that PTP-4 acts within the same cascade or functions through a parallel recognition route specifically triggered by PA14. The distinct phenotypes and binding partners identified in our study suggest that PTP-4 may provide an additional layer of immune modulation that is complementary to the functions of VHP-1. Subsequent studies have shown that mice deficient in the non-receptor-like tyrosine phosphatase PTP1B exhibit increased neutrophil infiltration and enhanced PA clearance in the lungs (Yue et al., 2016). Additionally, PTP1B can negatively regulate the MyD88/TRIF/IFN-1 signaling pathway, thereby influencing infection outcomes (Xu et al., 2008). SHP-1 negatively regulates IRAK1 to enhance the TLR/RIG-1/IFN-1 signaling pathway, modulating immune responses to infection (An et al., 2008). Mice lacking the PTPs protein PTPN2 in dendritic cells display increased infiltration of various immune cells, which is closely associated with the pathogenesis of inflammatory bowel disease (IBD). These studies demonstrate that protein tyrosine phosphatases (PTPs) play significant roles in immune regulation. Given the highly conserved structure of the PTP family, to explore whether other members of the PTP family also exert negative regulatory effects on infection immunity.

Our findings suggest that PTP-4 may function as a strategic checkpoint in the host immune network. While we demonstrated its role in negatively regulating PMK-1 phosphorylation, this could indirectly influence other p38-dependent or parallel pathways. Specifically, PTP-4-mediated signaling might modulate the activity of key transcriptional regulators such as ATF-7 (Fletcher et al., 2019), SKN-1 (Inoue et al., 2005), DAF-16 (Ding et al., 2023), or HLH-30 (Lee and Mylonakis, 2017). It is worth noting that several lines of evidence in our study support the predominance of the PMK-1 axis over these parallel defense systems. First, the resistance phenotype in *ptp-4* and *atln-1* mutants is specifically triggered by pathogen challenge without extending basal lifespan on *E. coli* OP50 (Figures 1, 3), a characteristic that distinguishes this axis from broader stress-response pathways like DAF-16 or SKN-1. Second, the robust elevation of p-PMK-1 in *ptp-4* mutants (Figure 2C) underscores a direct regulatory link to the p38 MAPK cascade. While we cannot entirely exclude the involvement of parallel pathways or ER stress sensors like XBP-1, given ATLN-1’s role in ER morphology—our data suggest that PMK-1 is the primary executor of this immunomodulatory effect. Future investigations utilizing stress-responsive reporters and candidate gene qPCR will be instrumental in fully mapping the potential cross-talk between the PTP-4/ATLN-1 axis and these parallel defense mechanisms. Therefore, the enhanced pathogen resistance in *ptp-4* mutants likely results from a broader optimized defense response rather than a singular signaling event. This underscores the potential of PTP-4 as a therapeutic target, where its inhibition could enhance multi-faceted host defenses against PA14 infection.

ATLN-1 is a key endoplasmic reticulum (ER) morphology-regulating GTPase belonging to the conserved GB1/RHD3 superfamily. Our phylogenetic analysis confirms that ATLN-1 functions as an ortholog of mammalian Atlastin (ATL) proteins (Sun et al., 2021; Zhao et al., 2020). Previous mechanistic studies have established that ATLs drive the homotypic fusion of ER tubules; importantly, this membrane remodeling activity is indispensable for critical cellular processes, including ER-phagy, autophagosome biogenesis, and the spatial organization of organelles (Liang et al., 2018; Rismanchi et al., 2008). While the involvement of ATLs in viral replication and host-virus interactions has been documented (Zlamalova et al., 2024; Shih and Hsueh, 2016; Zhang G. et al., 2023), their role in antibacterial innate immunity has remained largely unexplored. To functionally characterize the role of ATLN-1 in host defense, we challenged *C. elegans atln-1* loss-of-function mutants with *P. aeruginosa*. Notably, while the basal lifespan of these mutants on non-pathogenic *E. coli* OP50 was indistinguishable from that of wild-type animals (Figure 3C), *atln-1* deficiency conferred a robust and significant survival advantage during PA14 infection. This specific resistance phenotype indicates that ATLN-1 functions specifically under stress conditions rather than influencing general development or aging. Furthermore, the extended survival was strongly correlated with a marked reduction in intestinal bacterial burden, as evidenced by decreased colony-forming unit (CFU) counts (Figure 4D). This suggests that the loss of ATLN-1 potentiates the host’s ability to clear invading pathogens. Collectively, these findings for the first time established ATLN-1 as a novel negative regulator of pathogen resistance, highlighting an evolutionarily conserved mechanism where the balance of ER membrane homeostasis exerts a critical checkpoint effect on immune activation. The role of PTP-4/ATLN-1 as a negative regulator suggests that PA14 might hijack this existing host pathway to dismantle defenses from within. By stabilizing this endogenous “off-switch”, the pathogen suppresses immune activation to facilitate colonization, a tactic potentially targetable by pharmacological intervention.

Based on the elucidation of the PTP-4/ATLN-1 axis, we sought to explore pharmacological interventions targeting this pathway. We identified Baicalin, a bioactive flavonoid, as a potent inhibitor of PTP-4. Unlike previous studies that attributed the anti-inflammatory effects of Baicalin to broad-spectrum activities (He et al., 2021; Dinda et al., 2017), our work unveils a precise molecular mechanism: Baicalin binds directly to the PTP-4 protein (as revealed by SPR and docking), which subsequently promotes the dissociation of the PTP-4/ATLN-1 complex (as validated by Co-IP and site-directed mutagenesis), while simultaneously downregulating PTP-4 protein expression. This downregulation may stem from Baicalin-induced protein instability or enhanced degradation via the ubiquitin-proteasome system, a hypothesis that warrants further investigation (Banh et al., 2016). While the direct competitive disruption of the complex by Baicalin in a cell-free system remains to be further characterized by dual-protein SPR in future studies, our current multi-dimensional evidence—spanning *in silico* docking, *in vitro* direct binding, and cell-based interaction assays—consistently supports this regulatory model. However, since ATLN-1 is essential for maintaining ER membrane homeostasis, it is important to consider potential off-target or side effects of Baicalin on other ER-dependent intracellular processes. Nevertheless, our survival data on non-pathogenic *E. coli* OP50 suggest that Baicalin treatment at the tested concentrations does not compromise healthspan this “dual-hit” mechanism—allosteric inhibition of protein interaction and suppression of target abundance—parallels the phenotypes observed with the genetic loss of PTP-4, thereby enhancing host immune defenses against bacterial infection. Our results suggest that the PTP-4/ATLN-1 axis may represent a druggable target for anti-infective therapy.

In conclusion, our study elucidates a previously uncharacterized immune regulatory module regulated by the conserved PMK-1, PTP-4, and ATLN-1. We identified PTP-4 as a central checkpoint that orchestrates pathogen resistance through distinct mechanisms: modulating the canonical PMK-1 signaling pathway and physically interacting with the ER-shaping GTPase ATLN-1. By integrating genetic screening with pharmacological validation, we not only reveal ATLN-1 as a previously unrecognized immune regulator linking organelle dynamics to immunity but also establish Baicalin as a specific modulator of this axis. Consequently, targeting the PTP-4/ATLN-1 interaction represents a promising therapeutic strategy for enhancing host defense against distinct bacterial pathogens.

## Data Availability

The original contributions presented in the study are included in the article/Supplementary Material, further inquiries can be directed to the corresponding author.

## Supplementary material

SUPPLEMENTARY FIGURE S1 Schematic diagram of the PTP family members.

SUPPLEMENTARY FIGURE S2 The intracellular PTPase domain of PTP-4 shows high conservation with human PTP proteins. Sequence comparison of PTP motifs across human PTP family members and PTP-4 in *Caenorhabditis elegans*, coloured by percentage identity (Red). Amino acids are numbered according to the residue position in human PTPRA. The locations are shown at the top of the alignment. The PTP consensus motifs (M1 to M10) are detailed in the below of the alignment.

SUPPLEMENTARY FIGURE S3 The PTPase catalytic domain of PTPRA is highly conserved across multiple animal lineages. Multiple sequence alignments were performed on the intracellular PTPase domain of PTPRA (also known as PTPA) from brown rats (*Rattus norvegicus*), mice (*Mus musculus*), humans (*Homo sapiens*), and zebrafish (*Danio rerio*). Residue colors represent the percentage of sequence identity, with red shading indicating amino acids identical in all four species. The horizontal lines below the sequence represent ten typical PTP common motifs (motif 1 to motif 10). Amino acid numbering is consistent with the human PTPRA sequence. This alignment indicates that the catalytic core is almost completely evolutionarily conserved across multiple animal lineages, supporting its crucial role in tyrosine dephosphorylation.

SUPPLEMENTARY FIGURE S4 Loss of ptp-4 amplifies the transcriptional induction of innate immune effectors during P. aeruginosa PA14 infection.Transcriptional levels of **(A)** *clec-6*0 and **(B)** *lys-2* were determined by qRT-PCR in wild-type (N2) and *ptp-4*(*ok1411*) mutants exposed to either *E. coli* OP50 or PA14 for 12 h. Data were normalized to the expression of *gpd-2*. Data are presented as mean ± SD from three independent biological replicates. Group comparisons were conducted using two-way ANOVA followed by Tukey’s *post hoc* test. *P* < 0.05 was considered statistically significant (ns, not significant; ****, *P* < 0.0001).

SUPPLEMENTARY FIGURE S5 Prediction of the combination of PTP-4 and ATLN-1. **(A)** Schematic diagram of the predicted interaction structure of PTP-4 (AlphaFold: AF-P28192-F1, forest green) and ATLN-1(AlphaFold: AF-Q9BMU4-F1, cornflower blue). The color scheme is used in the below. **(B)** Interface summary of PTP-4 and ATLN-1(PDBePISA, https://www.ebi.ac.uk/msd-srv/prot_int/cgi-bin/piserver). **(C)** Cartoon representation of interaction interface: (a–d) Close-up view of interactions between PTP-4 and ATLN-1. The position of each interface is shown in the upper panel.

SUPPLEMENTARY FIGURE S6 Virtual screening pipeline and binding affinity analysis for PTP-4 inhibitors. **(A)** Schematic workflow of the systematic virtual screening. The screening utilized the COCONUT natural product database containing 715,344 molecules. The library underwent sequential filtering through a structure and toxicity filter, Lipinski’s “Rule of Five,” ADME (Absorption, Distribution, Metabolism, and Excretion) screening, and clustering/dereplication analysis. Finally, 1,054 representative compounds were subjected to molecular docking against the PTP-4 catalytic pocket, identifying Baicalin as the lead candidate. **(B)** Computational evaluation of candidate binding affinities. (Left) Frequency distribution of binding affinities (kcal/mol) for the screened compound library against PTP-4. The red dashed line indicates the position of Baicalin (ID: CNP0175313.1), which exhibits a superior binding energy of approximately −10.1 kcal/mol, ranking within the top 0.1% of the distribution. (Right) Ranking of the top 10 compounds based on their predicted binding affinities against the PTP-4 protein; Baicalin (highlighted in orange) emerged as a top-tier candidate with strong structural complementarity.
