# Supplementary figures and images for: Phosphatase PTP-4 downregulates ATLN-1-mediated host defense against Pseudomonas aeruginosa infection in Caenorhabditis elegans
Source: Front Pharmacol. 2026 Jun 1;17:1804420. doi: 10.3389/fphar.2026.1804420 (PMC13265298; doi:10.3389/fphar.2026.1804420)

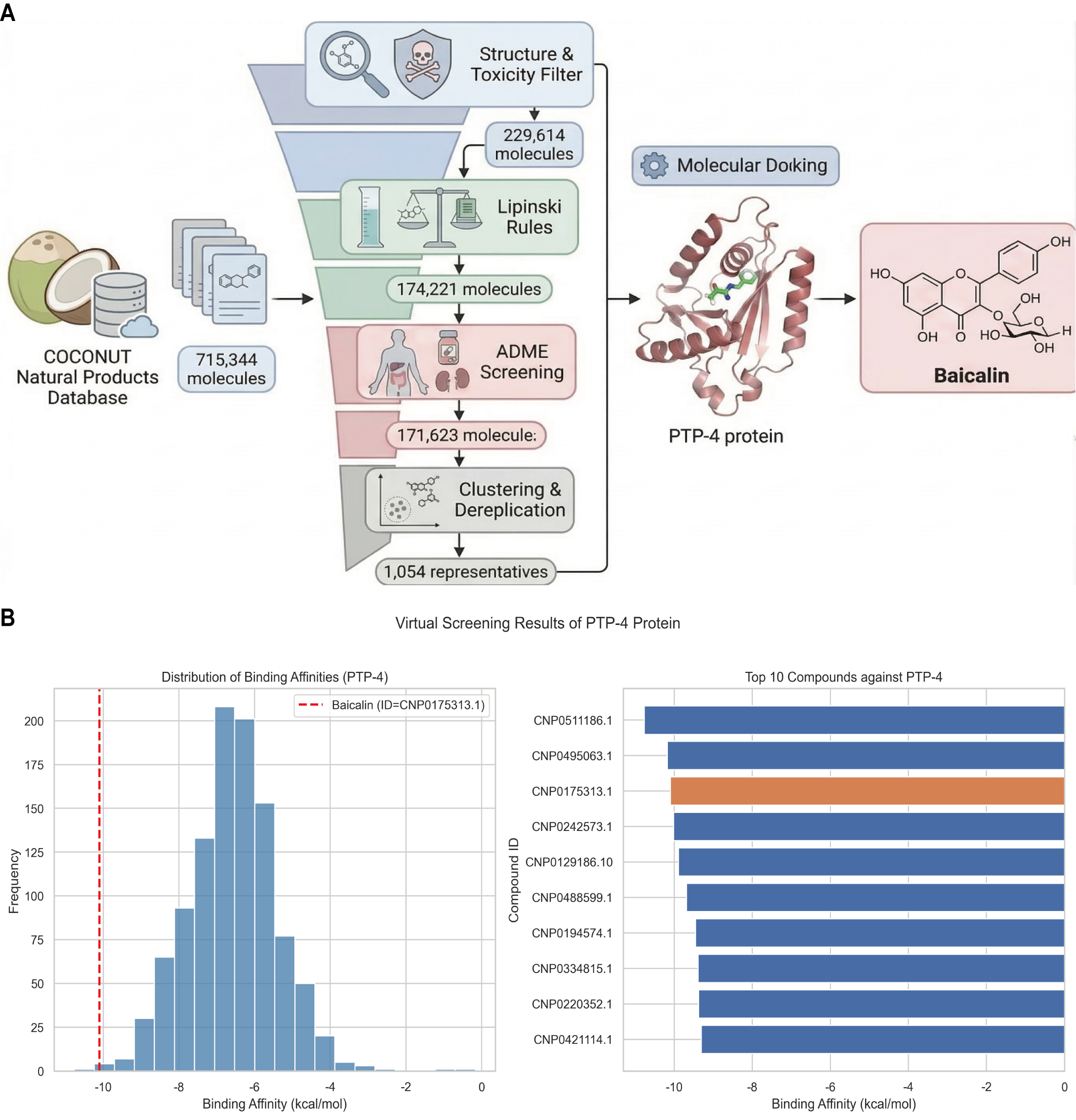

Supplement: Supplementary file 1 [file Image6.tif]

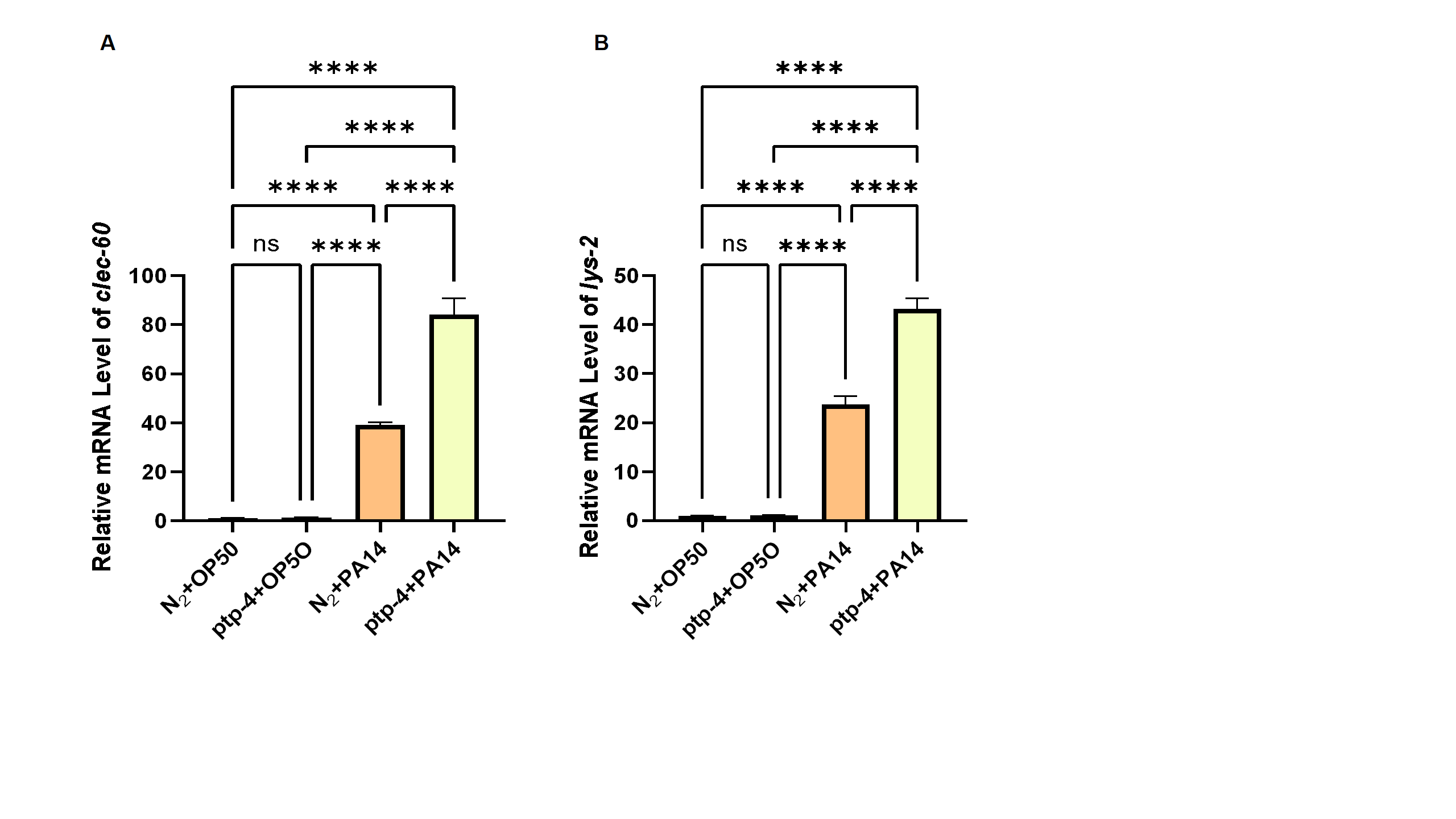

Supplement: Supplementary file 3 [file Image4.tif]

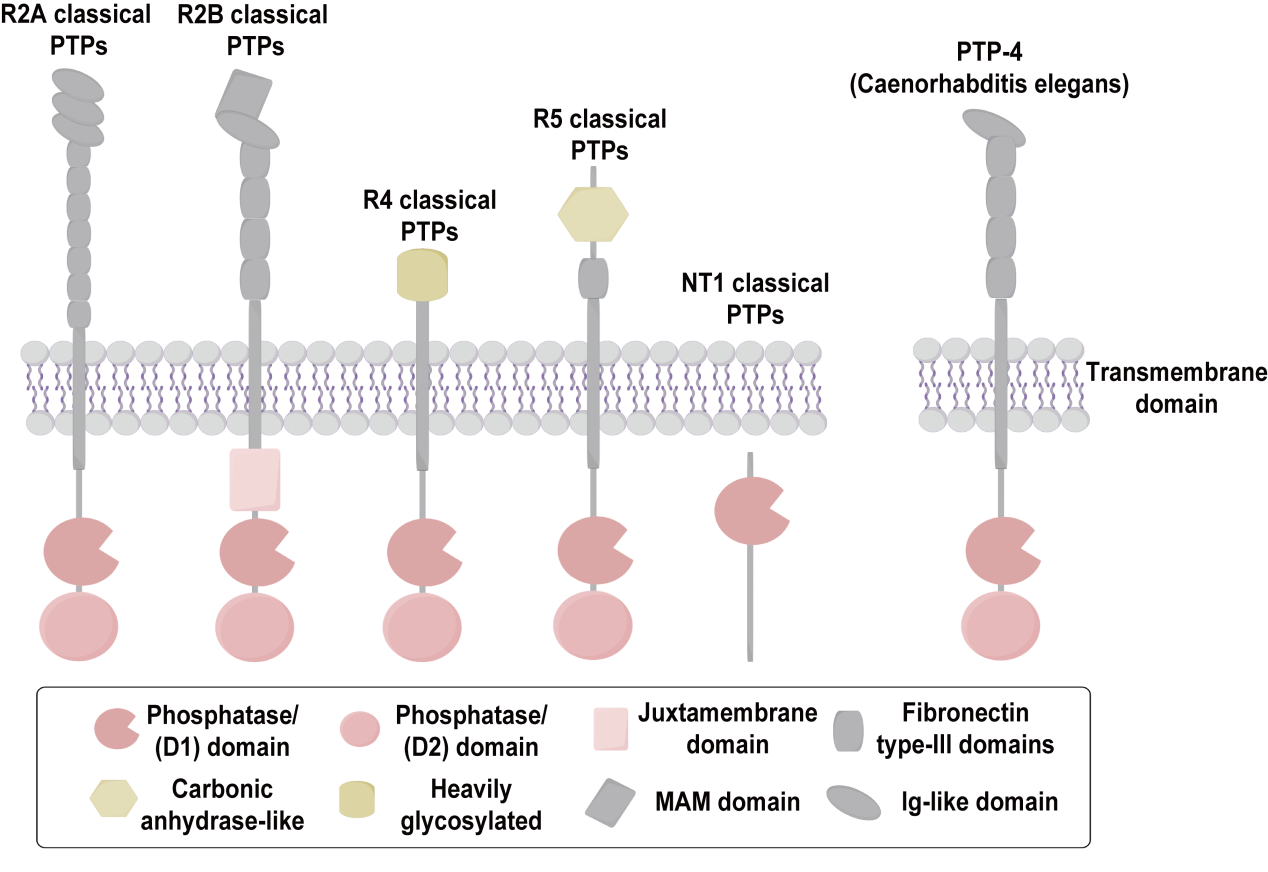

Supplement: Supplementary file 4 [file Image1.tif]

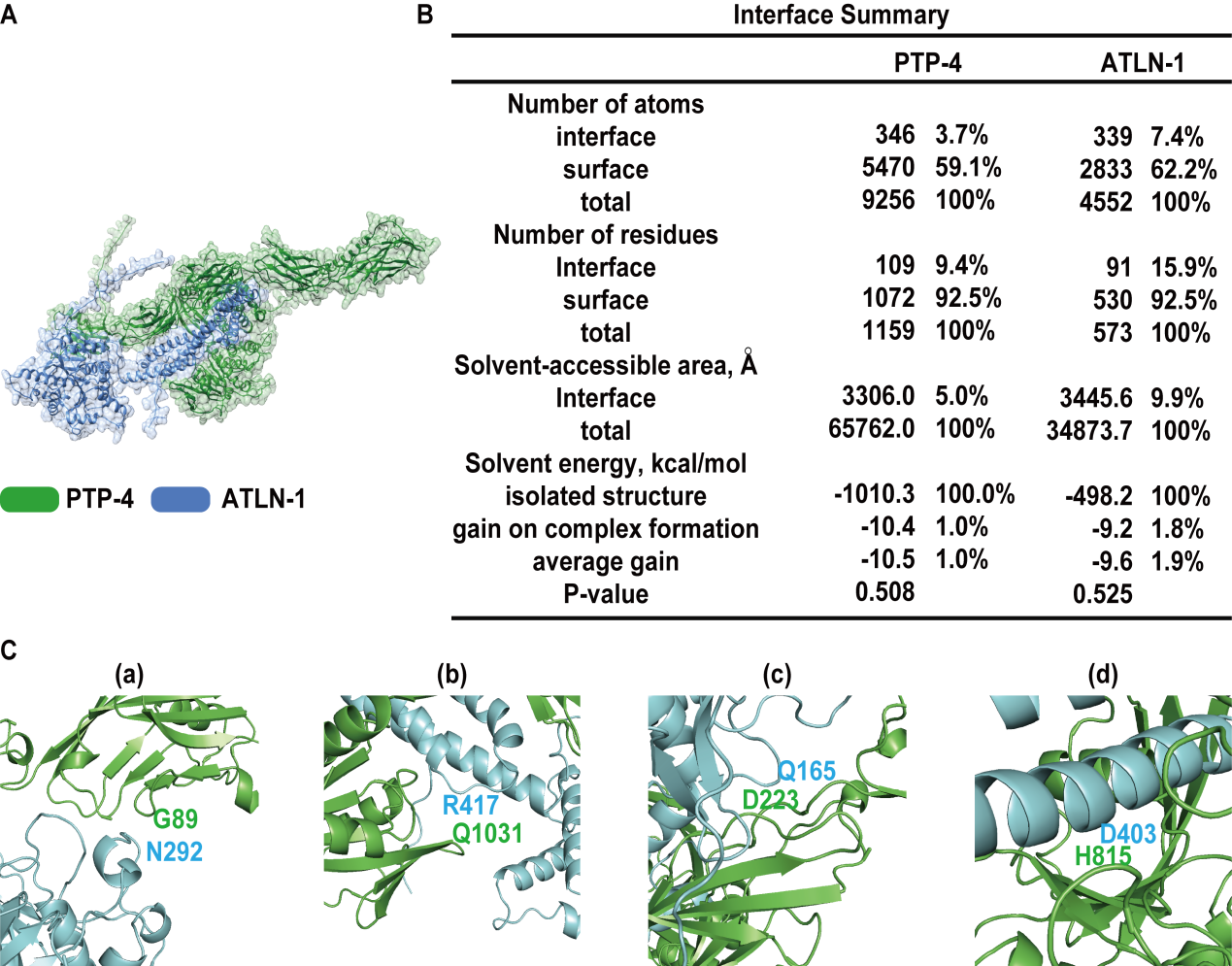

Supplement: Supplementary file 5 [file Image5.tif]

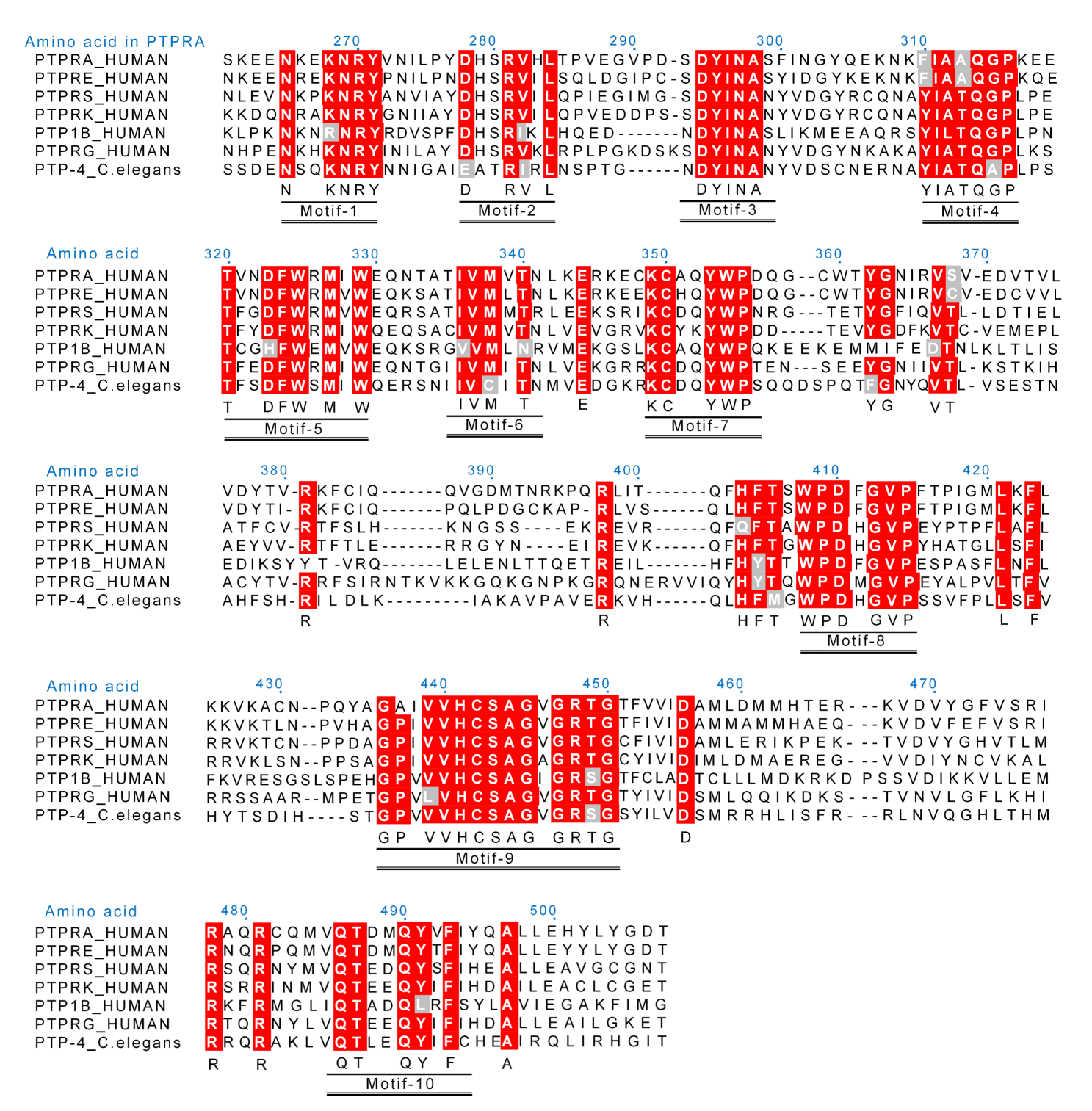

Supplement: Supplementary file 6 [file Image2.tiff]
